# PlantMolecularTasteDB: A Database of Taste Active Phytochemicals

**DOI:** 10.3389/fphar.2021.751712

**Published:** 2022-01-12

**Authors:** Teodora-Cristiana Gradinaru, Madalina Petran, Dorin Dragos, Marilena Gilca

**Affiliations:** ^1^ Department of Functional Sciences I/Biochemistry, Faculty of Medicine, Carol Davila University of Medicine and Pharmacy, Bucharest, Romania; ^2^ Department of Medical Semiology, Faculty of Medicine, Carol Davila University of Medicine and Pharmacy, Bucharest, Romania; ^3^ 1st Internal Medicine Clinic, University Emergency Hospital Bucharest, Carol Davila University of Medicine and Pharmacy, Bucharest, Romania

**Keywords:** taste, bitter, sweet, sour, pungent, astringent, phytochemicals, antiinflammatory

## 1 Introduction

In traditional medicine taste of medicinal plants represents one of the ethnopharmacological descriptors used for selection of the optimal herbal treatment of various ailments (Brett and Heinrich, 1998; Leonti et al., 2002; Gollin, 2004; Gilca and Barbulescu, 2015). Accumulating scientific evidence indicates that this ancient vision on the intrinsic therapeutic potency of herbal taste may not be completely devoid of a biological foundation (Gollin, 2004; Gilca and Dragos, 2017). Recent discoveries in taste science lead to the astonishing conclusion that the whole human body is endowed with a diffuse chemosensory system consisting of taste receptors and other chemesthesis sensors (Sbarbati and Osculati, 2005; Behrens and Meyerhof, 2011; Laffitte et al., 2014). This widespread extrasensorial expression of taste receptors and other oral chemosensors is increasingly recognized as a molecular basis for their non-gustative roles in many important biological processes, such as digestion (Harada et al., 2019), immune response (Douglas and Cohen, 2017), inflammation (Sharma et al., 2017), cell differentiation (Masubuchi et al., 2013), regulation of endocrine secretion (Clark et al., 2015), and many others (Behrens and Meyerhof, 2011; Laffitte et al., 2014).

Scientists have already suggested that these taste receptors and chemosensors might be druggable, thus having therapeutic potential (Lee et al., 2019). For instance, bitter taste receptors are considered potential critical players and therapeutic targets in inflammatory obstructive lung disease (Grassin-Delyle et al., 2019) and genito-urinary tract infections/inflammation (Welcome, 2020), while pungency chemosensor TRPA1 (transient receptor potential cation channel ankyrin 1) was proposed as a regulator of neurogenic inflammation (Logashina et al., 2019). This explains the recently renewed interest of scientific community for taste receptors and tastants.

Due to their great biodiversity, plants are a huge reservoir of taste active compounds characterized by an extreme chemical heterogeneity. More and more phytotastants and their biological roles mediated by taste receptors are discovered every year. Therefore, the development of a database focused on plant-derived tastants like PlantMolecularTasteDB valorizes this accumulating evidence and paves the way for a deeper understanding of the taste-related traditional medical epistemology.

PlantMolecularTasteDB is distinctive from other similar resources, such as BitterDB (Wiener et al., 2012; Dagan-Wiener et al., 2019) and SuperSweetDB (Ahmed et al., 2011), by focusing on the complex gustative profile of plant derived tastants/compounds (meaning combination of all five basic tastes and/or orosensations such as pungency, astringency, etc. characteristic for each given tastant/compound) and on their evidence-based anti-inflammatory activity (Table 1). Furthermore, PlantMolecularTasteDB is significantly richer in phytotastants than BitterDB and SuperSweetDB. It integrates data about all types of orosensorially active phytochemicals (not being focused on a single taste or orosensation).

**TABLE 1:** Comparison between various databases available on tastants and novelty of PlantMolecularTasteDB.

Feature	PlantMolecularTasteDB	BitterDB (Wiener et al., 2012)	SuperSweetDB (Ahmed et al., 2011)	FlavorDB (Garg et al., 2018)
Compounds	Bitter, sweet, sour, salty, umami, pungent and astringent compounds Complete taste profile for each phytochemical	Only bitter compounds	Only sweet tastants	No distinction between taste and odour of the compounds
	Focused on plant-derived tastants	Both natural and synthetic tastants	Both natural and synthetic tastants	Both natural and synthetic flavor molecules
Chemical class	Chemical class specified	–	Chemical class specified	Functional groups provided
Biological activity	Antiinflammatory activity	–	–	–

## 2 Database Overview

PlantMolecularTasteDB currently contains 1,527 phytochemicals that were reported in the literature as bitter (1,114 entries), sweet (263 entries), sour (61 entries), salty (7 entries), umami (25 entries), pungent (224 entries) or astringent (189 entries).

For each phytocompound PlantMolecularTasteDB offers information regarding synonyms, identifiers in international databases (PubChem ID, FooDB ID, HMDB ID, ChEMBL ID), molecular formula, chemical class, chemical structure, quantitative (taste threshold, where available) or qualitative sensorial data (taste/orosensorial profile, where available), affinity for taste receptors or chemosensors (both positive and negative evidence, threshold value, EC50, type of interaction-agonist/antagonist, activation/inhibition threshold value, where available), anti-inflammatory activity (both positive and negative evidence, where available), references with links for the phytochemical gustative properties and biological activity. Regarding the chemical classes, the best represented are the alkaloids (32%) and the terpenoids (21%).

The main categories of taste receptors and chemosensors from various species, targeted by the phytochemicals in PlantMolecularTasteDB are: a. bitter taste receptors (TAS2Rs); b. sweet taste receptor (TAS1R2/TAS1R3); c. umami taste receptor (TAS1R1/TAS1R3); d. potential candidates for sour taste receptors (ASICs, PKD2L1, mPKD2L1, KIR); e. chemosensors involved in pungency and other chemesthesis sensation (cooling, warmth, heat, irritation) (TRPVs); f. others (e.g., K^+^channels, Na^+^channels, GPR40, GPR120, GPR84).

PlantMolecularTasteDB was developed for researchers in the field of (ethno)pharmacology, taste sciences, nutrition. PlantMolecularTasteDB interface allows its users to perform simple or advanced searches and browsing the dataset. The phytochemicals can be searched by simple search options using the following criteria: name, identifiers, taste (or trigeminal orosensations), taste receptor (or chemosensor), chemical class, anti-inflammatory activity. The advanced search allows to retrieve phytochemicals by a combination of criteria (e.g., bitter AND astringent, sweet AND saponin, triterpene AND TAS2R14, astringent AND anti-inflammatory, TRPA1 AND antiinflammatory).

## 3 Materials and Methods

### 3.1 Data Aquisition

Data were collected from literature and publicly available databases. To begin with, a list of phytotastants was created using BitterDB (Wiener et al., 2012), SuperSweetDB (Ahmed et al., 2011), PubChem (https://pubchem.ncbi.nlm.nih.gov/), FooDB (https://foodb.ca/), PhytoMolTasteDB (Dragos and Gilca, 2018a). To this list, new taste active phytochemicals were added. They were identified by performing multiple systematic searches in PubMed, ScienceDirect and Google Scholar with various combinations of keywords: “phytochemical” AND “taste”, specific phytochemical name AND taste (e.g., “colubrine” AND “taste”), “phytochemical” AND (“bitter” OR “sweet” OR “sour” OR “salty” OR “astringent” OR “pungent” OR “umami”), specific phytochemical name AND specific taste (e.g., “colubrine” AND “bitter”), “phytochemical” AND specific taste receptor/chemosensor name (e.g., “phytochemical” AND “TAS2R”). To extract data of interest (e.g., sensorial description, taste thresholds) apart from electronic search, we have also performed intensive manual search, wherever required, from hundreds of publications.

We have included 730 bitter phytochemicals not yet included in BitterDB and 235 sweet phytochemicals not yet included in SuperSweetDB.

Regarding the data on taste, few of the main literature resources cited were: Bitterness in food and beverages by Rouseff (1990) (97 phytochemicals), Merck index (78 phytochemicals) (Smith et al., 2001), Food chemistry by Belitz et al. (2009) (48 phytochemicals).

Phytochemicals were included in the database if they fulfilled simultaneously two conditions: 1) they were reported as taste active (sensorially or experimentally through ligand -taste receptor affinity assays) or as capable to induce other orosensations, such as astringency or pungency; 2) their exact structures were available in PubChem, FooDB, HMDB, ChEMBL or at least in one scientific publication.

Phytochemical structures that were not found in PubChem, FooDB, HMDB, ChEMBL were drawn manually using MarvinSketch 21.7 ChemAxon, Inc. (https://chemaxon.com). Afterwards, canonical and isomeric SMILES not available in PubChem were generated using Chemicalize tool, ChemAxon (www.chemicalize.com).

We additionally introduced in our database few peculiar categories of taste inactive phytochemicals, such as bitter masking sakuranetin and jaceosidin (Fletcher et al., 2011; Roland et al., 2014), sweetness enhancer arabinogalactan (Kim and Kinghorn, 2002), few precursors or metabolites of phytotastants, e.g. arctigenin -not bitter (the aglycon of a bitter compound, arctiin) (Matsuo et al., 1972), S-1-propenyl-L-cysteine sulfoxide, S-methyl-L-cysteine sulfoxide (pungency precursors) (Sun Yoo and Pike, 1998).

An original feature of PlantMolecularTasteDB absent from other taste-focused databases consists of data on the evidence-based biological activity of the phytotastants. The first biological activity introduced in the present version of PlantMolecularTasteDB is the antiinflammatory activity. For this purpose, a systematic literature search was performed using the phrase: [specific phytochemical name] AND (antiinflammatory OR anti-inflammatory OR inflammation),” [e.g., azadirachtin AND (antiinflammatory OR anti-inflammatory OR inflammation)] in PubMed, Elsevier databases and Google Scholar. We aimed to collect at least two relevant studies (when available), regardless of experimental model (*in vitro*, animal study, clinical study), study design, language, year of publication or publication status. Standardized criteria were utilized for selection. Antiinflammatory activity was considered evidence-based if supported by at least one *in vitro*, animal or human study.

The references regarding the taste or the anti-inflammatory activity were categorized as “insufficient evidence” if we encountered one of the following situations:

1) Only one original old reference (dated before 1960) was available.
2) References derived from papers published in journals not included in PubMed, ScienceDirect or other prestigious international databases.
3) Only ambiguous results were available. For instance, for 4,2′-dihydroxychalcone, Roland et al. (2013) found ambiguous results for activation of both TAS2R14 and TAS2R39, when receptor assay was performed.
4) Only metabolomic profiling studies as references, e.g. kaempferol 3-O-p-coumaroyl-dirhamnosyl-glucoside (Zhu et al., 2017)

“Contradictory evidence” label was used if different authors reported opposite results, e.g., isovitexin reported as bitter (Zhu et al., 2017), non-bitter (Stark et al., 2005), and tasteless (Olennikov et al., 2015); capric acid reported as antiinflammatory (Lee and Kang, 2017) and also as proinflammatory (Tanaka et al., 2001).

Another original feature is a tool generating the so-called “Plant Molecular Taste” (PMT), which represents the virtual taste profile resulting from the contribution of all major orosensorially active phytocompounds found in the respective medicinal plant (Dragos and Gilca, 2018b). PMT is not necessarily the perceived taste, due to several reasons, including the quantitative one: a given phytotastant may be present in a certain plant only in a tiny amount, not surpassing its taste detection threshold. PMT was suggested to be a better predictor than the phytochemical class for the ethnopharmacological activities of the medicinal plants (Dragos and Gilca, 2018b). By convention, PMT graphical representation in PlantMolecularTasteDB takes into account each taste and trigeminal orosensation contribution to the gustative profile, calculated as a percentage of the total number of phytotastants present in that plant (or in a specific combination of phytochemicals).

### 3.2 Database Structure

#### 3.2.1 Web Server

PlantMolecularTasteDB was built as a relational database. It is hosted on a MariaDB type server version 5.5.47, protocol version 10. The website has a PHP server side. An Apache HTTP Server enables web access. The site is well adapted to all the most popular browsers, such as Chrome, Firefox, Opera, Edge, and Safari.

#### 3.2.2 Visualisation Tools

Marvin Sketch 21.7 plugin (http://www.chemaxon.com) enables the visualization of the molecular structure. PlantMolecularTasteDB contains a 2D compound structure display for each compound which is generated by Marvin JS 5.3.8, 2010 and a 3D rotation tool powered by ChemAxon (http://www.chemaxon.com). The chemical structure of phytochemicals can be downloaded as png or jpg.

## 4 Examples of Use

### 4.1 Searching for Sesquiterpenoid Lactones Which are Agonists of hTAS2Rs 3, 4, 5, 9, 10, 14, 30, 39, 40

These compounds are of research interest because both sesquiterpenoid lactones and agonists of the mentioned hTAS2Rs displayed anti-inflammatory potential in various experimental studies (Hall et al., 1979; Hohmann et al., 2016; Grassin-Delyle et al., 2019). The advanced search with the following keywords “sesquiterpenoid lactone” AND “[hTAS2R3] OR [hTAS2R4] OR [hTAS2R5] OR [hTAS2R9] OR [hTAS2R10] OR [hTAS2R14] OR [hTAS2R30] OR [hTAS2R39] OR [hTAS2R40]” yielded 9 compounds (3-beta-hydroxy dihydrocostunolide, 3-beta-hydroxypelenolide, absinthin, alpha-santonin, arglabin, artemorin, costunolide, parthenolide, picrotoxinin), while the more advanced search with the following keywords “sesquiterpenoid lactone” AND “[hTAS2R3] OR [TAS2R4] OR [TAS2R5] OR [TAS2R9] OR [hTAS2R10] OR [hTAS2R14] OR [TAS2R30] OR [TAS2R39] OR [TAS2R40]” AND “antiinflammatory” produced only 6 compounds (absinthin, alpha-santonin, arglabin, artemorin, costunolide, parthenolide). Therefore the 6 compounds may be investigated for the contribution of their TAS2R agonist quality to the anti-inflammatory activity, while the other three compounds (3-beta-hydroxy dihydrocostunolide, 3-beta-hydroxypelenolide, picrotoxinin) may be further investigated for a potential anti-inflammatory activity (mediated or not by TAS2Rs).

### 4.2 Searching for Selective or Common Agonists of Taste Receptors

In order to find selective agonists, the user should use the function Search → Advanced → Receptors all, and input the receptor of interest in the Agonist box, while the rest of the receptors in the Negative evidence box. For instance, 16 types of hTAS2Rs were found to be expressed in lung macrophages (Grassin-Delyle et al., 2015; Grassin-Delyle et al., 2019) and human bronchi (Grassin-Delyle et al., 2013), being involved in bitter agonist dependent-regulation of inflammation and bronchial relaxation. Therefore these TAS2Rs were proposed as new therapeutic targets in chronic obstructive lung diseases such as asthma (Grassin-Delyle et al., 2013; Grassin-Delyle et al., 2015). In order to find selective agonists for instance for hTAS2R46, the user should perform the following search: Agonist → [hTAS2R46] and Negative evidence → “{[hTAS2R3] AND [hTAS2R4] AND [hTAS2R5] AND [hTAS2R7] AND [hTAS2R8] AND [hTAS2R9] AND [hTAS2R10] AND [hTAS2R14] AND [hTAS2R19] AND [hTAS2R20] AND [hTAS2R31] AND [hTAS2R38] AND [hTAS2R39] AND [hTAS2R43] AND [hTAS2R45]}.” This search leads to two selective agonists for hTAS2R46, which are not recognized by the rest of the receptors: dehydroandrographolide and oxymatrine. Selective agonists for each TAS2R are of interest in order to find the specific functional roles for each type of receptor.

Common agonists for at least 2 types of receptors may also be of interest in order to identify potential functional synergy between them, which may be used to increase the pharmacological efficacy of a ligand. For instance, hTAS2R5, hTAS2R10, and hTAS2R14 expressed in bronchi had a predominant role in bitter agonist-induced bronchial relaxation (Grassin-Delyle et al., 2013). A double search in PlantMoleculaTasteDB 1) Search → Receptor→ “{[hTAS2R5] AND [hTAS2R10] AND [hTAS2R14]}”; 2) Search → Receptor→ “{[hTAS2R10] AND [hTAS2R14]}”), found no common agonist for all the three receptors, but 22 common agonists for hTAS2R10 and hTAS2R14.

### 4.3 Generating Plant Molecular Taste for a Medicinal Plant (Whole Composition or Partial Composition)

**FIGURE 1:**
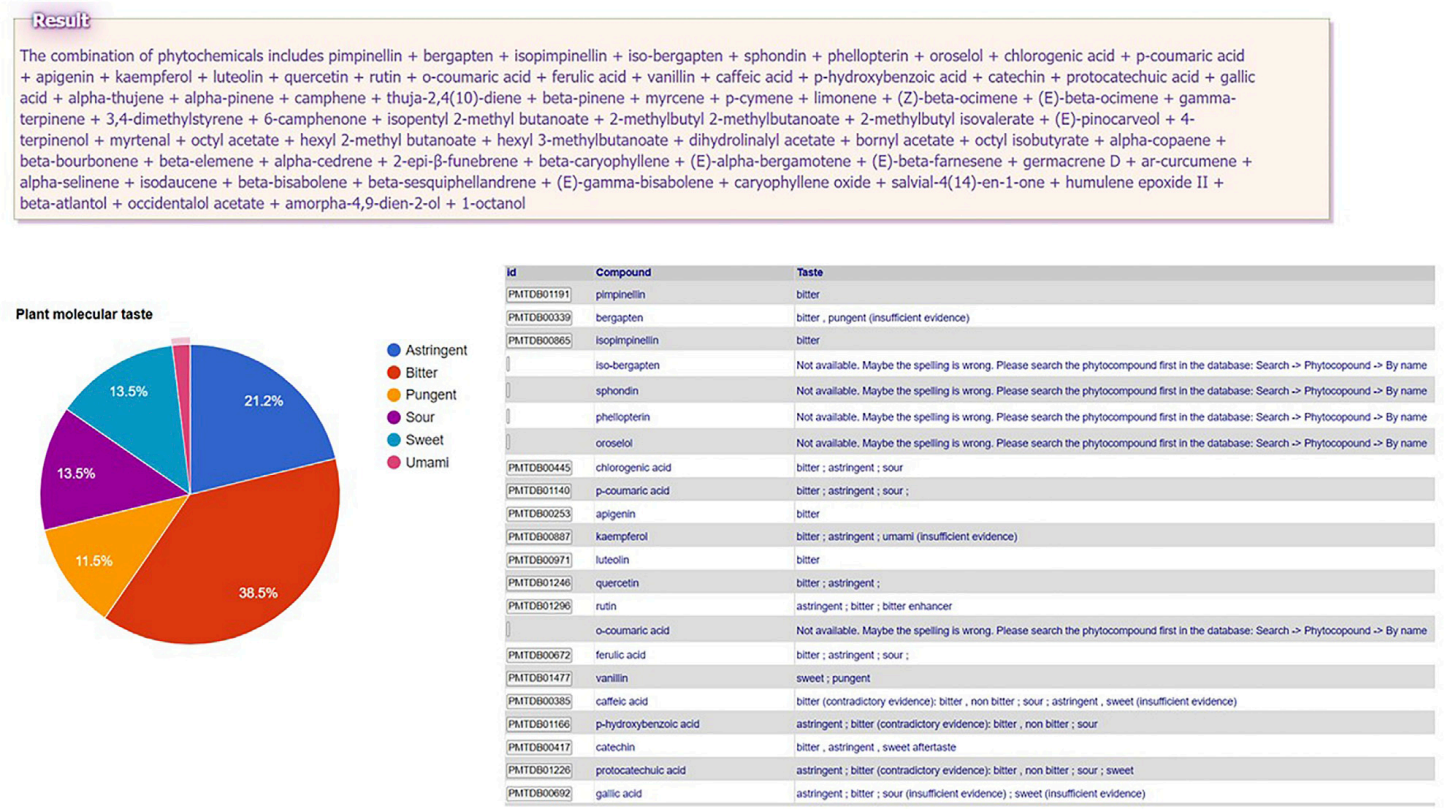
Plant Molecular Taste of small hogweed (*Heracleum sphondylium L*., Apiaceae family) generated in PlantMolecularTasteDB.

In order to obtain a PMT, the user need to upload the composition of that plant (the names of constituent phytochemicals separated by semicolons in the text area at the end of the menu path Tools → Plant Molecular Taste → Select compounds from PlantMolecularTasteDB list OR Paste the list of compounds. This tool is especially of interest for medicinal plants insufficiently studied, since PMT may predict some of the plant (ethno)pharmacological activities that are worth studying experimentally (Gilca and Barbulescu, 2015; Dragos and Gilca, 2018a; Dragos and Gilca, 2018b). For instance, small hogweed (*Heracleum sphondylium L*., Apiaceae family) is traditionally used to treat several human ailments (e.g. flatulence, stomachache, diarrhea, epilepsy, hypertension, wounds, menstrual problems, impotence) (Bahadori et al., 2016), but is insufficiently studied. A search in PubMed with the keyword “*Heracleum sphondylium*” leads to only 21 papers, showing some evidence regarding only its antioxidant, antimutagenic, antimicrobial, and vasorelaxant activities. The phytochemical profile is relatively documented, the plant being rich in essential oil (Matejic et al., 2016), phenolic compounds (Uysal et al., 2019), and furanocoumarins (Dresler et al., 2018). The user has the possibility to upload: A) the entire list of the known major constituent phytochemicals of this plant (pimpinellin; bergapten; isopimpinellin etc.—see the box “Result” in Figure 1 for the complete list) or B) the composition of partial extracts (e.g., only essential oil constituents). The whole PMT is bitter > astringent > sweet = sour > pungent > umami (Figure 1). The essential oil molecular taste is pungent = bitter > sweet. Interestingly, pungency, which is an organoleptic characteristic of essential oils in general, is traditionally associated with antiinfectious and antiparasitic activity, which were proven for *Heracleum sphondylium* essential oil by some experimental studies (Matejic et al., 2016; Uysal et al., 2019). Bitter taste was suggested as a predictor of antiinflammatory activity (Dragos and Gilca, 2018b; Dragos et al., 2021), while bitter taste receptors TAS2Rs showed recently anti-inflammatory effects (Sharma et al., 2017; Grassin-Delyle et al., 2019). The role of *Heracleum sphondylium* L as a source of inflammation modulating agents is yet to be explored.

## 5 Limitations of PlantMolecularTasteDB

1) Not all the phytochemicals found in our database were investigated for their anti-inflammatory potential, therefore lack of any information marked by “No evidence” at the rubric Antiinflammatory activity means either negative evidence (true lack of anti-inflammatory activity) or lack of studies evaluating the anti-inflammatory activity of that compound.
2) Only a few physicochemical features (only molecular formula, chemical structure and chemical class) are available in the present version of PlantMolecularTasteDB.
3) The generation of PMT is dependent on the limited number of phytotastants existent in PlantMolecularTasteDB and on the scarcity of data regarding the chemical composition of certain medicinal plants.
4) Some of the phytochemicals submitted for generating PMT and declared “not found” may actually be present in PlantMolecularTasteDB but under a different name (orthographic variant or synonym). As a workaround for this issue the user might either choose from the list of phytocompounds (Tools → Plant Molecular Taste → Select compounds from PlantMolecularTasteDB) (which however does not include synonyms) or look for each phytochemical declared “not found,” as the dedicated tool (Search → Phytocompound → By name) also explores the synonyms.

## 6 Conclusion

PlantMolecularTasteDB is the first database dedicated to all types of orosensorially active phytochemicals (bitter, sweet, sour, umami, salty, pungent, astringent phytochemicals). Its novelty over the other similar databases resides in the focus on the plant derived tastants, information related to sourness, pungency, astringency, complete taste profile, and antiinflammatory activity of the phytotastants. According to our knowledge, it is the database that contains the highest number of phytotastants and orosensation active phytochemicals. PlantMolecularTasteDB now provides a unique platform for further studies aiming to predict sensorial profile of unlisted phytocompounds or potential ligands for TASRs or TRPs. PlantMolecularTasteDB will be regularly upgraded with new phytotastants, physico-chemical features, biological activities, plants sources and interactive tools [e.g., taste predictive tool of newly discovered (phyto)chemicals].

## Data Availability

PlantMolecularTasteDB is openly available at www.plantmoleculartastedb.org. Further inquiries can be directed to the corresponding author.

## Abbreviations

ASIC, acid-sensing ion channels; PMT, Plant Molecular Taste; TASR, taste receptor; TRP, transient receptor potential; TRPA1, transient receptor potential cation channel ankyrin 1; TRPC, Transient receptor potential canonical; TRPM, transient receptor potential melastatin; TRPV, transient receptor potential vanilloid.

## References

[B1] Ahmed J. Preissner S. Dunkel M. Worth C. L. Eckert A. Preissner R. (2011). SuperSweet--a Resource on Natural and Artificial Sweetening Agents. Nucleic Acids Res. 39, D377–D382. 10.1093/nar/gkq917 20952410 PMC3013782

[B2] Bahadori M. B. Dinparast L. Zengin G. (2016). The Genus Heracleum: a Comprehensive Review on its Phytochemistry, Pharmacology, and Ethnobotanical Values as a Useful Herb. Compr. Rev. Food Sci. Food Saf. 15, 1018–1039. 10.1111/1541-4337.12222 33401836

[B3] Behrens M. Meyerhof W. (2011). Gustatory and Extragustatory Functions of Mammalian Taste Receptors. Physiol. Behav. 105, 4–13. 10.1016/j.physbeh.2011.02.010 21324331

[B4] Belitz H. D. Grosch W. Schieberle P. (2009). Food Chemistry. 4th ed. Leipzig: Springer Berlin Heidelberg. 10.1007/978-3-540-69934-7

[B5] Brett J. A. Heinrich M. (1998). Culture, Perception and the Environment the Role of Chemosensory Perception. Angew. Bot. 72, 67–69.

[B6] Clark A. A. Dotson C. D. Elson A. E. Voigt A. Boehm U. Meyerhof W. (2015). TAS2R Bitter Taste Receptors Regulate Thyroid Function. FASEB J. 29, 164–172. 10.1096/fj.14-262246 25342133 PMC4285546

[B7] Dagan-Wiener A. Di Pizio A. Nissim I. Bahia M. S. Dubovski N. Margulis E. (2019). BitterDB: Taste Ligands and Receptors Database in 2019. Nucleic Acids Res. 47, D1179–D1185. 10.1093/nar/gky974 30357384 PMC6323989

[B8] Douglas J. E. Cohen N. A. (2017). Taste Receptors Mediate Sinonasal Immunity and Respiratory Disease. Int. J. Mol. Sci. 18, 437. 10.3390/ijms18020437 PMC5343971 28218655

[B9] Dragos D. Gilca M. (2018b). Taste of Phytocompounds: A Better Predictor for Ethnopharmacological Activities of Medicinal Plants Than the Phytochemical Class? J. Ethnopharmacol 220, 129–146. 10.1016/j.jep.2018.03.034 29604378

[B10] Dragos D. Gilca M. (2018a). PhytoMolecularTasteDB: An Integrative Database on the "molecular Taste" of Indian Medicinal Plants. Data in Brief 19, 1237–1241. 10.1016/j.dib.2018.04.048 30246068 PMC6141601

[B11] Dragos D. Petran M. Gradinaru T. Gilca M. (2021). “P134. Do Bitter Phytocompounds Target Inflammation-Related Macromolecules,” in The 20th International Congress of International Society for Ethnopharmacology, Virtual Congress, Greece, April 18th – 20th, 247.

[B12] Dresler S. Bogucka-Kocka A. Kováčik J. Kubrak T. Strzemski M. Wójciak-Kosior M. (2018). Separation and Determination of Coumarins Including Furanocoumarins Using Micellar Electrokinetic Capillary Chromatography. Talanta 187, 120–124. 10.1016/j.talanta.2018.05.024 29853023

[B13] Fletcher J. N. Kinghorn A. D. Slack J. P. McCluskey T. S. Odley A. Jia Z. (2011). *In Vitro* Evaluation of Flavonoids from Eriodictyon Californicum for Antagonist Activity against the Bitterness Receptor hTAS2R31. J. Agric. Food Chem. 59, 13117–13121. 10.1021/jf204359q 22059530 PMC4391372

[B14] Gilca M. Barbulescu A. (2015). Taste of Medicinal Plants: a Potential Tool in Predicting Ethnopharmacological Activities? J. Ethnopharmacol. 174, 464–473. 10.1016/j.jep.2015.08.040 26320686

[B15] Gilca M. Dragos D. (2017). Extraoral Taste Receptor Discovery: New Light on Ayurvedic Pharmacology. Evid. Based Complement. Alternat Med. 2017, 5435831–5435930. 10.1155/2017/5435831 28642799 PMC5469997

[B16] Gollin L. X. (2004). Subtle and Profound Sensory Attributes of Medicinal Plants Among the Kenyah Leppo’ Ke of East Kalimantan, Borneo. J. Ethnobiol. 24, 173–201. Available at: http://direct.biostor.org/reference/175093.

[B17] Grassin-Delyle S. Abrial C. Fayad-Kobeissi S. Brollo M. Faisy C. Alvarez J. C. (2013). The Expression and Relaxant Effect of Bitter Taste Receptors in Human Bronchi. Respir. Res. 14, 134. 10.1186/1465-9921-14-134 24266887 PMC4176101

[B18] Grassin-Delyle S. Naline E. Devillier P. (2015). Taste Receptors in Asthma. Curr. Opin. Allergy Clin. Immunol. 15, 63–69. 10.1097/ACI.0000000000000137 25486380

[B19] Grassin-Delyle S. Salvator H. Mantov N. Abrial C. Brollo M. Faisy C. (2019). Bitter Taste Receptors (TAS2Rs) in Human Lung Macrophages: Receptor Expression and Inhibitory Effects of TAS2R Agonists. Front. Physiol. 10, 1267. Available at: https://www.frontiersin.org/article/10.3389/fphys.2019.01267. 10.3389/fphys.2019.0126710.3389/fphys.2019.01267 31632299 PMC6783802

[B20] Hall I. H. Lee K. H. Starnes C. O. Sumida Y. Wu R. Y. Waddell T. G. (1979). Anti-inflammatory Activity of Sesquiterpene Lactones and Related Compounds. J. Pharm. Sci. 68, 537–542. 10.1002/jps.2600680505 311831

[B21] Harada Y. Koseki J. Sekine H. Fujitsuka N. Kobayashi H. (2019). Role of Bitter Taste Receptors in Regulating Gastric Accommodation in Guinea Pigs. J. Pharmacol. Exp. Ther. 369, 466–472. 10.1124/jpet.118.256008 30967403

[B22] Hohmann M. S. N. Longhi-Balbinot D. T. Guazelli C. F. S. Navarro S. A. Zarpelon A. C. Casagrande R. (2016). “Sesquiterpene Lactones,” in Studies in Natural Products Chemistry (Amsterdam, Boston, Heidelberg, London, New York, Oxford, Paris, San Diego, San Francisco, Singapore, Sydney, Tokyo: Elsevier), 243–264. 10.1016/b978-0-444-63601-0.00007-7

[B23] Kim N.-C. Kinghorn A. D. (2002). “Sweet-tasting and Sweetness-Modifying Constituents of Plants. Studies in Natural Products Chemistry. 27, 3. 10.1016/S1572-5995(02)80033-3

[B24] Laffitte A. Neiers F. Briand L. (2014). Functional Roles of the Sweet Taste Receptor in Oral and Extraoral Tissues. Curr. Opin. Clin. Nutr. Metab. Care 17, 379–385. 10.1097/MCO.0000000000000058 24763065 PMC4059820

[B25] Lee S. I. Kang K. S. (2017). Function of Capric Acid in Cyclophosphamide-Induced Intestinal Inflammation, Oxidative Stress, and Barrier Function in Pigs. Sci. Rep. 7, 16530. 10.1038/s41598-017-16561-5 29184078 PMC5705592

[B26] Lee S. J. Depoortere I. Hatt H. (2019). Therapeutic Potential of Ectopic Olfactory and Taste Receptors. Nat. Rev. Drug Discov. 18, 116–138. 10.1038/s41573-018-0002-3 30504792

[B27] Leonti M. Sticher O. Heinrich M. (2002). Medicinal Plants of the Popoluca, México: Organoleptic Properties as Indigenous Selection Criteria. J. Ethnopharmacol. 81, 307–315. 10.1016/S0378-8741(02)00078-8 12127230

[B28] Logashina Y. A. Korolkova Y. V. Kozlov S. A. Andreev Y. A. (2019). TRPA1 Channel as a Regulator of Neurogenic Inflammation and Pain: Structure, Function, Role in Pathophysiology, and Therapeutic Potential of Ligands. Biochemistry (Mosc) 84, 101–118. 10.1134/S0006297919020020 31216970

[B29] Masubuchi Y. Nakagawa Y. Ma J. Sasaki T. Kitamura T. Yamamoto Y. (2013). A Novel Regulatory Function of Sweet Taste-Sensing Receptor in Adipogenic Differentiation of 3T3-L1 Cells. PLoS One 8, e54500. 10.1371/journal.pone.0054500 23336004 PMC3545961

[B30] Matejic J. S. Dzamic A. M. Mihajilov-Krstev T. Ristic M. S. Randelovic V. N. Krivošej Z. Ð. (2016). Chemical Composition, Antioxidant and Antimicrobial Properties of Essential Oil and Extracts fromHeracleum sphondyliumL. J. Essent. Oil Bearing Plants 19, 944–953. 10.1080/0972060X.2014.986538

[B31] Matsuo K. Tokoroyama T. Kubota T. (1972). Bitter Constituents of Forsythia Viridissima. Phytochemistry 11, 1522–1523. 10.1016/s0031-9422(00)90135-3

[B32] Olennikov D. N. Kashchenko N. I. Chirikova N. K. Koryakina L. P. Vladimirov L. N. (2015). Bitter Gentian Teas: Nutritional and Phytochemical Profiles, Polysaccharide Characterisation and Bioactivity. Molecules 20, 20014–20030. 10.3390/molecules201119674 26556333 PMC6331966

[B33] Roland W. S. Gouka R. J. Gruppen H. Driesse M. van Buren L. Smit G. (2014). 6-Methoxyflavanones as Bitter Taste Receptor Blockers for hTAS2R39. PLoS One 9, e94451. 10.1371/journal.pone.0094451 24722342 PMC3983201

[B34] Roland W. S. van Buren L. Gruppen H. Driesse M. Gouka R. J. Smit G. (2013). Bitter Taste Receptor Activation by Flavonoids and Isoflavonoids: Modeled Structural Requirements for Activation of hTAS2R14 and hTAS2R39. J. Agric. Food Chem. 61, 10454–10466. 10.1021/jf403387p 24117141

[B35] Rouseff R. L. (1990). Bitterness in Foods and Beverages. Netherlands: Elsevier Amsterdam.

[B36] Sbarbati A. Osculati F. (2005). The Taste Cell-Related Diffuse Chemosensory System. Prog. Neurobiol. 75, 295–307. 10.1016/j.pneurobio.2005.03.001 15882778

[B37] Sharma P. Yi R. Nayak A. P. Wang N. Tang F. Knight M. J. (2017). Bitter Taste Receptor Agonists Mitigate Features of Allergic Asthma in Mice. Sci. Rep. 7, 46166. 10.1038/srep46166 28397820 PMC5387415

[B38] Smith A. Heckelman P. E. Obenchain J. R. Gallipeau J. A. R. D’Arecca M. A. Budavari S. (2001). The Merck Index. Thirteenth Edition. Whitehouse Station. New Jersey, USA: Merck Co., Inc.

[B39] Stark T. Bareuther S. Hofmann T. (2005). Sensory-Guided Decomposition of Roasted Cocoa Nibs (Theobroma Cacao) and Structure Determination of Taste-Active Polyphenols. J. Agric. Food Chem. 53, 5407–5418. 10.1021/jf050457y 15969527

[B40] Sun Yoo K. Pike L. M. (1998). Determination of flavor precursor compound s-alk(en)yl-L-cysteine sulfoxides by an HPLC method and their distribution in allium species. Scientia Horticulturae 75, 1–10. 10.1016/S0304-4238(98)00107-1

[B41] Tanaka S. Saitoh O. Tabata K. Matsuse R. Kojima K. Sugi K. (2001). Medium-chain Fatty Acids Stimulate Interleukin-8 Production in Caco-2 Cells with Different Mechanisms from Long-Chain Fatty Acids. J. Gastroenterol. Hepatol. 16, 748–754. 10.1046/j.1440-1746.2001.02537.x 11446882

[B42] Uysal A. Ozer O. Y. Zengin G. Stefanucci A. Mollica A. Picot-Allain C. M. N. (2019). Multifunctional Approaches to Provide Potential Pharmacophores for the Pharmacy Shelf: Heracleum Sphondylium L. Subsp. Ternatum (Velen.) Brummitt. Comput. Biol. Chem. 78, 64–73. 10.1016/j.compbiolchem.2018.11.018 30500554

[B43] Welcome M. O. (2020). The Bitterness of Genitourinary Infections: Properties, Ligands of Genitourinary Bitter Taste Receptors and Mechanisms Linking Taste Sensing to Inflammatory Processes in the Genitourinary Tract. Eur. J. Obstet. Gynecol. Reprod. Biol. 247, 101–110. 10.1016/j.ejogrb.2020.02.015 32088528

[B44] Wiener A. Shudler M. Levit A. Niv M. Y. (2012). BitterDB: a Database of Bitter Compounds. Nucleic Acids Res. 40, D413–D419. 10.1093/nar/gkr755 21940398 PMC3245057

[B45] Zhu M. Li N. Zhao M. Yu W. Wu J. L. (2017). Metabolomic Profiling Delineate Taste Qualities of tea Leaf Pubescence. Food Res. Int. 94, 36–44. 10.1016/j.foodres.2017.01.026 28290365

